# Accuracy of migraine diagnosis and treatment by neurologists in the Baltic states: e-survey with clinical case challenge

**DOI:** 10.1186/s40001-023-01555-z

**Published:** 2023-12-18

**Authors:** Mantas Jokubaitis, Greta Vrublevska, Linda Zvaune, Mark Braschinsky, Alo-Rainer Leheste, Gatis Saknītis, Danils Žukovs, Kristina Ryliškienė

**Affiliations:** 1 Centre of Neurology, Vilnius University, Santariškių St. 2, 08406 Vilnius, Lithuania; 2 Institute of Clinical Medicine, Vilnius University, Vilnius, Lithuania; 3 Department of Neurology and Neurosurgery, Riga East Clinical University Hospital, Riga, Latvia; 4 Department of Neurology and Neurosurgery, Tartu University Hospital, Tartu, Estonia; 5 Neurology Clinic, University of Tartu, Tartu, Estonia; 6 Faculty of Medicine, Riga Stradins University, Riga, Latvia; 7 Department of Neurology, Pauls Stradins Clinical University Hospital, Riga, Latvia

**Keywords:** Accuracy, Migraine, Diagnosis, Treatment, Burden, Prophylaxis, Anti-CGRP, Triptans

## Abstract

**Background:**

Underdiagnosis of migraine causes a significant health burden, including lower quality of life, excessive medication use, and a delay in effective treatment. The purpose of this study was to evaluate migraine diagnosis accuracy and to review the treatment approaches used by neurologists in the Baltic states.

**Methods:**

The research was conducted as an anonymous e-survey with four cases in March and April 2021.

**Results:**

119 practicing adult neurologists have participated. The migraine diagnostic accuracy was 63.2%. The most commonly used diagnostic criteria were moderate/severe pain, unilateral pain, and disruption of daily activities. Diagnostic accuracy did not differ significantly between neurologists who always use ICHD-3 criteria and those who don’t (68.4% vs. 58.5%, *p* = 0.167). It was higher in neurologists who were working in headache centers (91.7% vs. 60.9%, *p* = 0.012), and was related to a higher percentage of migraine diagnoses in all consulted headache patients (*R*^2^ = 0.202, adjusted *R*^2^ = 0.195, *p* < 0.001), prophylaxis with onabotulinumtoxin A [OR = 4.332, 95% Cl (1.588–11.814)], and anti-CGRP monoclonal antibodies [OR = 2.862, 95% Cl (1.186–6.907)].

**Conclusions:**

Migraine diagnostic accuracy is improved through practical patient counseling and modern treatment prescription. Although the neurologists in the Baltic states follow current European guidelines, there is room for improvement in diagnostic accuracy to reduce migraine burden.

**Supplementary Information:**

The online version contains supplementary material available at 10.1186/s40001-023-01555-z.

## Background

There are many up-to-date resources available to learn how to recognize migraine effectively, including international [1] and national guidelines, and other easy-to-use recommendations [2]. According to the Global Burden of Diseases study, 3.07 billion people worldwide suffer from headaches, with migraine accounting for 43.37% of the total [3]. Nevertheless, in many parts of the world, migraine remains underdiagnosed and undertreated [4, 5]. In one Italian study, out of 953 migraine patients referred to headache center only 26.8% had a previous migraine diagnosis [6]. In another study conducted in the United States, 80% of 2991 sinusitis-related headache patients were instead diagnosed with migraine and 8% fulfilled diagnostic criteria for possible migraine diagnosis during the study [7]. Moreover, only 12.4% of 266 migraine patients were previously diagnosed with migraine in the Russian survey [8]. Reasons for insufficient migraine diagnosis include low awareness among the patients [9], insufficient knowledge of migraine between the doctors [10, 11], a lack of biological migraine markers, and the patient history being the sole basis of migraine diagnosis [1]. In addition, it has been demonstrated that headache subspecialization is not highly prestigious or attractive among neurologists [12–14]. Insufficient migraine diagnosis results in poor quality of life, economic burden, medication overuse and increases the risk of chronic migraine, comorbidities, and treatment refractoriness [15, 16]. Furthermore, it leads to a delay in seeking out available modern treatment [17].

Despite the fact that general practitioners should be able to identify and treat the majority of migraine cases, in the Baltic states migraine diagnosis is most commonly made by a neurologist. Furthermore, the Baltic countries lack specialized headache clinics, and access to modern migraine treatment varies greatly between the states. For example, Lithuania is unique in that the state fully reimburses two monoclonal antibodies against calcitonin-gene-related peptide (CGRP) or its receptor (fremanezumab, erenumab), allowing for effective migraine treatment, whereas onabotulinumtoxin A treatment for chronic migraine is only reimbursed in Estonia. In addition, even acute migraine treatment is not reimbursed in Latvia.

The three major gaps in headache care quality include overutilization of neuroimaging, underuse of preventive therapies, and inappropriate acute headache treatment. Lifting The Burden (LTB) and European Headache Federation (EHF) developed a set of headache service quality indicators, with accurate diagnosis being one of the nine major quality domains required for optimal headache care [18]. The panel of experts has formulated a multidimensional definition of quality of headache care: “good quality care achieves accurate diagnosis and individualized management, has appropriate referral pathways, educates patients about their headaches and their management, is convenient and comfortable, satisfies patients, is efficient and equitable, assesses outcomes and is safe.” [18, 19].  As part of a collaborative project between LTB and EHF, the service quality evaluation program tested these nine indicators in specialized headache centers and at the primary care level while also establishing standards of excellence for specialized headache centers [20]. The research has revealed that diagnostic inaccuracies were the most prevalent in primary care, where a significant proportion of patients received non-specific ICD-10 codes such as R51 (“headache”) rather than specific headache diagnoses. In contrast, ICHD-3 terminology was used in more than 90% of cases in specialized headache centers [18]. An analogous situation was observed with the recording of headache histories (especially time profiles) and the use of headache calendars and diaries, which were more accurate in specialized headache centers than in primary care [20].

The aim of this study was to compare the answers to theoretical questions about diagnostic criteria and their actual application in the four clinical cases that were presented to assess the accuracy of migraine diagnosis and to review the treatment approaches employed by neurologists in the Baltic states. The study's hypothesis was that practical migraine patient counseling and modern treatment prescription, rather than theoretical knowledge of International Classification of Headache Disorders third edition (ICHD-3) criteria, improves migraine diagnostic accuracy.

## Methods

We designed a questionnaire for this cross-sectional study that included the following sections: (1) demographic information (age, sex, occupation, years of practice, workplace type), (2) headache diagnosis-related information (the number of patients with headache in general and migraine among all consultations, the use of ICHD-3 criteria [1], types of migraine that one finds difficult to diagnose), (3) four clinical cases (with provided five most likely diagnoses, and single choice answer), and (4) questions related to the use of pharmacological and non-pharmacological migraine treatment and evaluation of its efficacy. The full questionnaire is presented in Additional file 1. The four clinical cases (two cases of migraine without aura, one case of migraine with aura, and one case of chronic migraine with medication overuse headache) were created following diagnostic criteria of ICHD-3 by a panel of three highly experienced headache specialists. The first clinical case of migraine without aura included all four migrainous headache characteristics according to ICHD-3 (unilateral, pulsating, moderate intensity, aggravated by physical activity) followed by one accompanying symptom (photo-/phonophobia). The second clinical case of migraine without aura included only two migrainous headache characteristics i.e., the bare minimum required for migraine diagnosis (severe intensity and aggravation by physical activity) followed by one accompanying symptom (nausea). The third clinical case of migraine with aura included typical aura symptoms (gradually appearing and fully reversible visual, sensory, and language symptoms with a total duration of 1 h) followed by non-migrainous headache or no headache. The fourth clinical case of chronic migraine with medication overuse headache included a typical duration and frequency of headache (almost every day, being severe 15 days per month, for many years), three migrainous headache characteristics (pulsating, severe intensity, aggravated by physical activity), one accompanying symptom (nausea) and the overuse of non-opioid painkiller combination with caffeine (15 days per month).

An advisory panel of neurologists from each Baltic State (Estonia, Latvia, Lithuania) assessed the initial survey. Some small improvements were made during the assessment. A pilot version was completed by a group of 10 neurologists with no further adjustments. The final anonymous online questionnaire was distributed through various neurology organizations (Lithuanian Society of Neurology, Estonian Headache Society, Estonian Ludvig Puusepp Society of Neurologists and Neurosurgeons, Latvian Society of Neurology). Participants were included in the study if they were neurologists currently practicing adult neurology. The study was conducted in the Baltic States from March 12 to April 13, 2021.

The survey completion was voluntary, and no financial incentive was received by the study participants. None of the involved organizations received any funding for the distribution of the survey. Since the data acquired was anonymous and without the ability to identify a specific person, no ethics approval was sought. Ethics approval was deemed unnecessary by Vilnius Regional Biomedical Research Ethics Committee with respect to the General Data Protection Regulation Principle 26. All participants consented to participate by marking confirmation in the e-survey that they agree to the use of their anonymous data for scientific publication. According to Article 2 of the Republic of Lithuania's Law on Ethics of Biomedical Research, no particular informed consent was necessary, as affirmed by the Vilnius Regional Biomedical Research Ethics Committee, since anonymous surveys are not considered biomedical research.

### Statistical analysis

Values were expressed as counts and frequencies for qualitative variables and as means or medians for quantitative variables, depending on the normality of the distribution. A statistical comparison between the three Baltic countries was done. Categorical variables were compared using the *χ*^2^ test (Pearson’s Chi-square or Fisher’s exact test when appropriate). The Kruskal–Wallis H test was used to compare non-parametric variables. Linear regression analysis was applied to identify the associations between independent quantitative variables and the diagnostic accuracy of migraine. We calculated unadjusted and adjusted *R*^2^, *p* values. The relationship between the independent categorical variables and correct answers in all clinical cases was assessed with logistic regression analysis. Odds ratios (OR), 95% confidence intervals (CI), and *p* values were computed. All statistical analyses were performed using IBM SPSS Statistics for Windows, V.20.0. For all comparisons, *p* < 0.05 was considered statistically significant.

## Results

In total, 119 neurologists participated in the study, of them 76 (63.9%) from Lithuania, 30 (25.2%) from Latvia and 13 (10.9%) from Estonia. The proportion of neurologists who participated in the study compared to all neurologists with an active license at the time of the study was 17.7% (76/429) in Lithuania, 15.6% (30/192) in Latvia, and 10.2% (13/128) in Estonia. 74.8% of participants were female, the median age of participants was 52.0 ± 13 years (27–74), and the median duration of work experience was 22.0 ± 14 years (1–52). 88.2% of all participants worked in an outpatient clinic, 7.6% of all neurologists worked in a specialized headache center. Estonian neurologists worked in specialized headache centers more frequently (30.8%, *p* = 0.004). Neurologists' experience with migraine diagnosis in their clinical practice is presented in Table 1.

**Table 1:** Neurologists' experience with migraine diagnosis

	Total	Lithuania	Latvia	Estonia	*p* value
Over 50 headache patients consulted per month	8.4% (*N* = 10)	6.6% (*N* = 5)	10.0% (*N* = 3)	15.4% (*N* = 2)	0.358
Over 50% of all headache patients have migraine diagnosis	18.5% (*N* = 22)	17.1% (*N* = 13)	20.0% (*N* = 6)	23.1% (*N* = 3)	0.851
Neurologists always using ICHD-3 criteria for migraine diagnosis	47.9% (*N* = 57)	50.0% (*N* = 38)	40.0% (*N* = 12)	53.8% (*N* = 7)	0.586

*ICHD-3* the third international classification of headache disorders

**Fig. 1:**
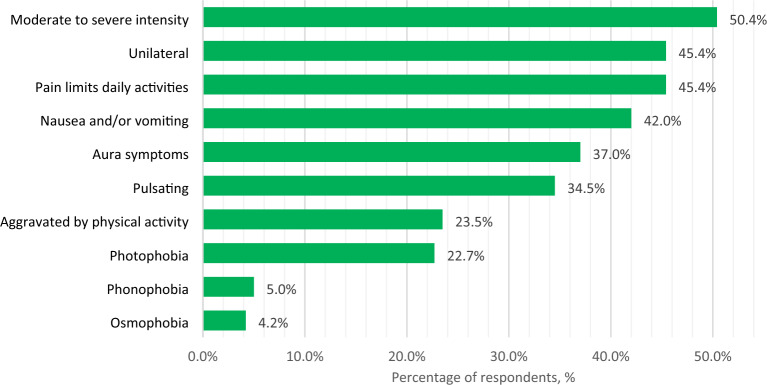
Most common migraine diagnostic criteria used by respondents. *Respondents were asked to choose the three most commonly used diagnostic criteria

**Fig. 2:**
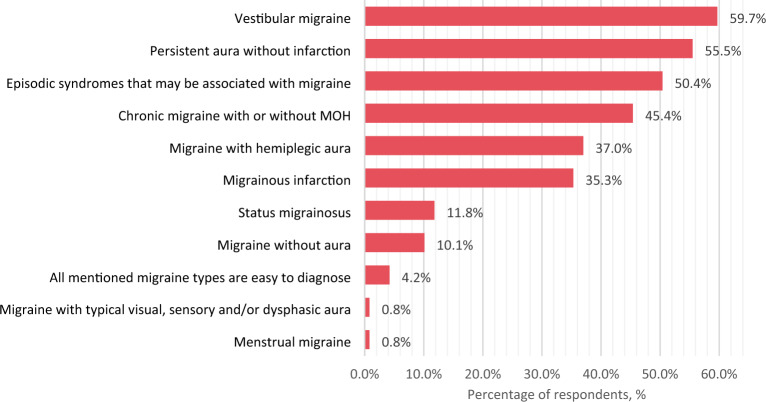
Difficult-to-diagnose migraine types as rated by respondents. *MOH* medication overuse headache

The three most commonly used migraine diagnostic criteria in clinical practice were: moderate/severe pain, unilateral pain, and disruption of daily activities (Fig. 1). Respondents indicated that vestibular migraine, persistent aura without infarction, episodic syndromes that may be associated with migraine, and chronic migraine were the most difficult-to-diagnose migraine types (Fig. 2).

There was a significant difference in the use of onabotulinumtoxin A and anti-CGRP monoclonal antibodies for prophylactic treatment of migraine between the Baltic states (Table 2).

**Table 2:** Neurologists’ experience with acute and prophylactic treatment

	Total	Lithuania	Latvia	Estonia	*p* value
Triptan use in over 50% of migraine patients	58.8% (*N* = 70)	60.5% (*N* = 46)	50.0% (*N* = 15)	69.2% (*N* = 9)	0.441
Oral migraine prophylaxis prescribed in over 50% of migraine patients	26.9% (*N* = 32)	30.3% (*N* = 23)	20.0% (*N* = 6)	23.1% (*N* = 3)	0.532
Use of neuromodulation*	22.7% (*N* = 27)	18.4% (*N* = 14)	33.3% (*N* = 10)	23.1% (*N* = 3)	0.256
Use of onabotulinumtoxin A for chronic migraine	17.6% (*N* = 21)	9.2% (*N* = 7)	13.3% (*N* = 4)	76.9% (*N* = 10)	< 0.001
Use of anti-CGRP monoclonal antibodies	39.5% (*N* = 47)	48.7% (*N* = 37)	20.0% (*N* = 6)	30.8% (*N* = 4)	0.020

*CGRP* calcitonin gene-related peptide

^*^External trigeminal nerve stimulation or noninvasive vagal nerve stimulation

**Fig. 3:**
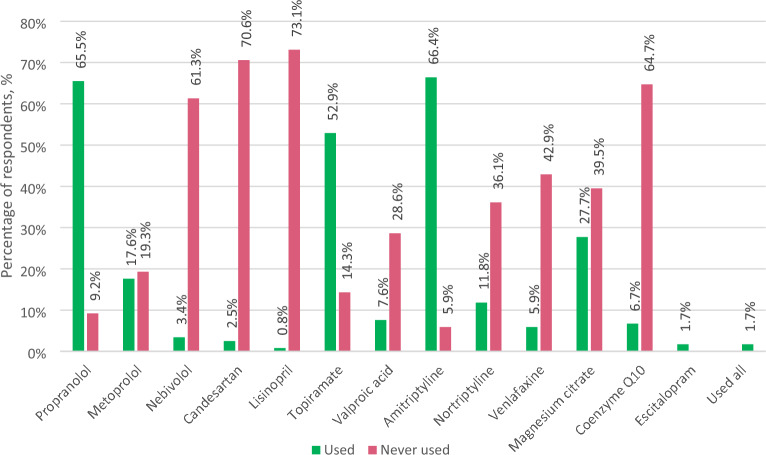
Most commonly used and never used oral migraine prophylaxis. *Respondents were asked to choose the three most commonly used and three never used prophylactic drugs or supplements

**Fig. 4:**
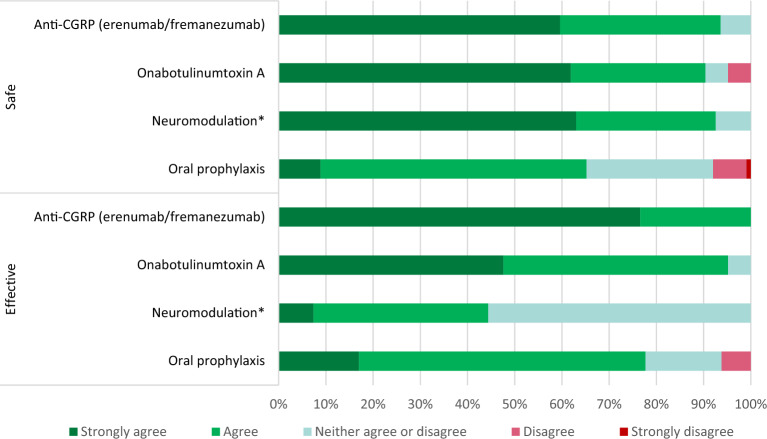
Neurologists’ opinion about the efficacy and safety of oral migraine prophylaxis, neuromodulation, onabotulinum toxin A and anti-calcitonin gene-related peptide (CGRP) treatment (erenumab, fremanezumab). *External trigeminal nerve stimulation or non-invasive vagal nerve stimulation

The most commonly used migraine oral prophylaxis drugs were propranolol, amitriptyline, and topiramate, whereas lisinopril, candesartan, and coenzyme Q10 were the most commonly listed as never used (Fig. 3). Figure 4 depicts respondents' perception of the safety and efficacy of prophylactic migraine treatment.

The average number of correct clinical case answers was 2.53 ± 1.10. The total migraine diagnostic accuracy was found to be 63.2% (301/476), see Table 3.

**Table 3:** Diagnostic accuracy of migraine clinical cases by neurologists of the Baltic states

	Total	Lithuania	Latvia	Estonia	*p* value
Migraine without aura, 1st case	87.4% (*N* = 104)	88.2% (*N* = 67)	83.3% (*N* = 25)	92.3% (*N* = 12)	0.698
Migraine without aura, 2nd case	42.0% (*N* = 50)	43.4% (*N* = 33)	33.3% (*N* = 10)	53.8% (*N* = 7)	0.42
Migraine with typical aura, 3rd case	60.5% (*N* = 72)	61.8% (*N* = 47)	53.3% (*N* = 16)	69.2% (*N* = 9)	0.572
Chronic migraine with MOH, 4th case	63.0% (*N* = 75)	68.4% (*N* = 52)	33.3% (*N* = 10)	100% (*N* = 13)	< 0.001
All cases answered correctly	22.7% (*N* = 27)	28.9% (*N* = 22)	3.3% (*N* = 1)	30.8% (*N* = 4)	0.014
Number of correct answers	63.2% (301/476)	65.5% (199/304)	50.8% (61/120)	78.9% (41/52)	0.016

*MOH* medication overuse headache

A diagnostic accuracy was found to be significantly higher in neurologists who are working in specialized headache centers (91.7% vs. 60.9%, *p* = 0.012), diagnosing migraine in more than 50% of their headache patients (78.4% vs. 59.8%, *p* = 0.009), prescribing triptans (64.8% vs. 25.0%, *p* = 0.009), prescribing oral migraine prophylaxis (64.7% vs. 39.3%, *p* = 0.043). Diagnostic accuracy did not differ significantly between neurologists who always use ICHD-3 diagnostic criteria for migraine diagnosis and those who do not (68.4% vs. 58.5%, *p* = 0.167).

Linear regression models showed that the number of correct diagnoses was related to the percentage of migraine diagnoses in all consulted headache patients (*R*^2^ = 0.202, adjusted *R*^2^ = 0.195, *p* < 0.001), the younger age of neurologist (*R*^2^ = 0.092, adjusted *R*^2^ = 0.085, *p* = 0.001), and the shorter clinical work experience (*R*^2^ = 0.104, adjusted *R*^2^ = 0.096, *p* < 0.001). Significant logistic regression findings related to correct answers in all clinical cases included the use of onabotulinumtoxin A [OR = 4.332, 95% Cl (1.588–11.814)], the prescription of neuromodulation treatment [OR = 2.595, 95% Cl (1.011–6.657)], the prescription of anti-CGRP treatment [OR = 2.862, 95% Cl (1.186–6.907)] and the use of disability and/or other headache-specific scales in clinical practice [OR = 3.266, 95% Cl (1.278–8.344)].

## Discussion

This is the first study to explore the diagnostic accuracy of migraine between neurologists in the Baltic States. While we must acknowledge the lack of validation, one of the strengths of our research is that it evaluates a practical approach to migraine diagnosis through clinical case solution rather than merely testing knowledge of ICHD-3 criteria. In addition, we collected data on the prescription of preventive treatment in daily practice and assessed neurologists' perspectives on migraine prevention. The hypothesis of our research has been confirmed as the diagnostic accuracy of migraine did not differ significantly between neurologists who always use ICHD-3 diagnostic criteria and those who do not (68.4% vs. 58.5%, *p* = 0.167). Nevertheless, despite being statistically insignificant, the difference of almost 10% may still be important clinically. The number of correct clinical case diagnoses was related to the higher percentage of migraine diagnoses in all consulted headache patients, the use of onabotulinumtoxin A, neuromodulation, and the prescription of anti-CGRP treatment. To the best of our knowledge, there have been no analogous studies in the literature concerning the migraine diagnostic accuracy by neurologists. Somewhat similar studies included physical therapists [21] and general practitioners [22, 23]. Nevertheless, the aforementioned studies used significantly different case vignettes and/or answer structures, and the presented diagnostic accuracy among different specialties cannot be compared.

The overall diagnostic accuracy of migraine among neurologists was found to be 63.2%. The diagnostic accuracy ranged from 50.8% in Latvia, to 65.5% in Lithuania and 78.9% in Estonia. The higher accuracy of Estonian neurologists could be explained by the fact that the majority of respondents worked in a more specialized headache center. Only 18.5% of respondents in their clinical practice diagnose migraine in over 50% of their headache patients. The small proportion of migraine diagnosis in everyday practice reflects the discrepancy between the actual migraine prevalence and the number of clinically diagnosed migraine. Therefore, a paradigm that has been formulated back in 2002 by Sheftell and Tepper should be remembered by many: “[…] episodes of disabling headache, with a stable pattern over years, should be viewed as migraine until proven otherwise.” [24].

In our study, the three most common symptoms used to diagnose migraine were pain intensity, location (unilateral), and headache impact on daily activities. Only one of these symptoms (disruption of daily activities) is used in “ID Migraine”—a validated migraine screening tool (the other two being nausea and photophobia) [25]. It was found by meta-analysis that “ID Migraine” is characterized by pooled sensitivity of 0.84 and specificity of 0.76 [26], whereas in a validation study [25], headache intensity and unilateral location were the two least specific symptoms for a migraine diagnosis. Nevertheless, the statement of consistent use of ICHD-3 criteria for migraine diagnosis was not found to be significantly associated with higher migraine diagnostic accuracy in our study. The responses regarding the use of the ICHD-3 criteria and the selection of the most important clinical criteria for migraine diagnosis partially contradicted the clinical case answers. The first case of migraine without aura had the highest diagnostic accuracy (87.4%), as it met all four diagnostic pain criteria. In contrast, 58% of neurologists did not recognize the second case's severe headache and associated vomiting as a migraine, most likely due to the lack of pulsating quality and unilateral location. Consequently, in the real world, such patients suffering from severe headache attacks would be left without migraine diagnosis and appropriate treatment. Another discrepancy can be seen between the second and fourth cases: while respondents indicated that chronic migraine was more difficult to diagnose than migraine without aura, the diagnostic accuracy of chronic migraine was higher in presented clinical cases. In summary, the use of clinical case challenges has the potential to increase the likelihood of detecting diagnostic errors. However, because there was no comparator group in this study, this conclusion is speculative.

Triptans are rarely used in the Baltic countries. Although a lack of reimbursement may account for the lower percentage of triptan use in Latvia, an analysis of the remaining data suggests that triptans might be preferentially used for severe attacks only. Propranolol, amitriptyline, and topiramate were the most commonly used migraine oral prophylaxis drugs by neurologists in the Baltic states. The use of these drugs is in accordance with the latest, but still in need of updating, European Federation of Neurological Societies' migraine treatment guidelines [27]. As many as 70.6% of neurologists indicated they had never prescribed candesartan, one of the most commonly prescribed drugs in Scandinavian countries near the Baltic States, and 42.9% indicated they had never prescribed venlafaxine. These two drugs were shown to be effective in migraine prevention much later than propranolol, amitriptyline, and topiramate. Therefore, such result could be attributed to a lack of latest knowledge about preventative migraine treatment.

Finally, different drug reimbursement laws between the Baltic states may have resulted in disparities in modern preventive treatment. All respondents rated anti-CGRP monoclonal antibodies as not only the most effective, but also one of the safest migraine treatments. While Lithuanian neurologists have extensive experience with erenumab and fremanezumab due to drug reimbursement for several years now, numerous studies, including meta-analyses [28, 29], have also confirmed the efficacy and safety of anti-CGRP treatment. On the other hand, neuromodulation had received the most neutral answers in both characteristics of safety and efficacy. In authors’ opinion, this could be explained by the lower availability (no reimbursement in any of the Baltic states), novelty of external trigeminal nerve stimulation and non-invasive vagal nerve stimulation, and thus a lack of knowledge and experience with this treatment approach.

There are several limitations to our research. Firstly, merely 10 to 17% of neurologists with active licenses in the respective Baltic state participated in the study. The small number of respondents, as well as non-response bias due to the nature of electronic surveys (older respondents and those with limited knowledge of information technology may have been excluded), may significantly limit the generalizability of our study's findings. Secondly, since no additional diagnoses had to be provided in clinical cases, closed-ended questions could have resulted in greater diagnostic accuracy. In addition, no feedback questions were asked in the survey, i.e., respondents were not questioned as to why the diagnosis of migraine was not selected, which would aid in the more accurate preparation of educational materials for neurologists. Finally, we did not use a validated data collection method, and the clinical case creation was limited to an expert panel.

## Conclusion

This is the first study to explore the diagnostic accuracy of migraine and treatment used by neurologists in the Baltic states. The results of our research show that the treatment used by neurologists complies with the latest European guidelines on effective migraine treatment. Nevertheless, to improve the quality of life of migraine patients, it is not enough for neurologists to know what treatment should be prescribed—a sufficient number of patients must also receive it. To achieve this goal, it is necessary to improve migraine diagnostic accuracy as well as to increase the use of effective acute and preventive migraine treatment.

### Supplementary Information

**Additional file 1.**Complete survey of the study.

## Data Availability

The datasets used and/or analysed during the current study are available from the corresponding author on reasonable request.
