# Supplementary material for: Accuracy of migraine diagnosis and treatment by neurologists in the Baltic states: e-survey with clinical case challenge
Source: Eur J Med Res. 2023 Dec 18;28:600. doi: 10.1186/s40001-023-01555-z (PMC10726575; doi:10.1186/s40001-023-01555-z)
Supplement: Supplementary file 1 — Additional file 1.Complete survey of the study. [file 40001_2023_1555_MOESM1_ESM.pdf]

Additional file 1. Complete survey of the study.

1. Country of residence:

- Lithuania
- Latvia
- Estonia

2. Age ___________________

3. Gender:

- Female
- Male

4. Specialty:

- Neurologist -> 4.1, 4.2, 4.3
- Child neurologist -> 4.1, 4.2, 4.3
- Neurology resident ->
- Child neurology resident ->

Indicate the year of your residency studies

- 1
- 2
- 3
- 4
- 5
- 6

4.1 -> Years of practice ___________________________________

4.2 -> What type of health institution do you work at (*select all that apply*):

- Secondary/tertiary health care centre
- Headache centre/clinic
- Private medical centre

4.3 -> What type of health institution/department do you work at (*select all that apply*):

- Outpatient clinic
- Inpatient hospital
- Emergency department

5. What percentage of your consulted patients are headache patients?

- 0%
- 1-10%
- 11-25%
- 26-50%
- 51-75%
- 76-90%
- 91-100%

6. On average, how many headache patients do you consult every month?

- 0
- 1-10
- 11-20
- 21-50
- 51-75
- 76-100
- > 100

7. For what proportion of your headache patients do you diagnose migraine?

- 0%
- 1-10%
- 11-25%
- 26-50%
- 51-75%
- 76-90%
- 91-100%

8. Do you follow the migraine diagnostic criteria of the International Classification of Headache Disorders on a daily basis?

- Yes, always
- Yes, often
- Yes, but rarely
- I do not
- I am unfamiliar with such criteria

9. Which 3 criteria, in your opinion, are the most important for migraine diagnosis? Choose from the list below.

- Unilateral headache
- Pulsating headache
- Moderate to severe pain intensity
- Headache is aggravated by routine physical activity (walking or climbing stairs)
- Headache limits everyday activities (functional disability)
- Headache is accompanied with nausea and/or vomiting
- Headache is accompanied with sensitivity to light (photophobia)
- Headache is accompanied with sensitivity to sounds (phonophobia)
- Headache is accompanied with sensitivity to odors (osmophobia)
- Aura symptoms

10. Which types of migraine do you find difficult to diagnose? (select all that apply)

- Migraine without aura
- Migraine with typical visual, sensory, and/or dysphasic aura
- Migraine with hemiplegic aura
- Chronic migraine with or without medication overuse headaches
- Vestibular migraine
- Menstrual migraine
- Status migrainosus
- Migrainous infarction

- Persistent aura without infarction
- Episodic Syndromes that may be associated with migraine
- Diagnosis of all listed migraine types is not difficult
- Other

Medical history is an essential part of the diagnosis of primary headaches (incl. tension-type headache and migraine). We present 4 cases of patients suffering from headaches, please indicate ONE preliminary diagnosis that seems most likely to you..

11. The 28-year-old female is consulted for episodic headache for about 10 years, with a frequency of 1 episode per month. The headache is pulsating, located on the right side of the head. The headache is of moderate intensity (4/10). It is associated with a slight sensitivity to sound and light, there is no nausea and vomiting. The headache is aggravated by routine physical activity. If untreated, the headache lasts for 1 day. The patient has no other known diseases. Physical examination and brain imaging is normal. Indicate the most likely diagnosis:

- Tension-type headache
- Psychogenic headache
- Migraine without aura
- Migraine with aura
- Other -> Please specify ____________

12. The 21-year-old female is consulted for episodic headaches for about 4 years, with a frequency of 2-3 episodes per month. The holocephalic headache is pressing, severe (7/10), aggravated by routine physical activity, no photo-/phonophobia is present, the pain is accompanied with slight nausea without vomiting. Pain lasts for 4 hours after taking ibuprofen 400 mg. The patient has no other known diseases. Physical examination and brain imaging is normal. Indicate the most likely diagnosis:

- Tension-type headache
- Psychogenic headache
- Migraine without aura
- Migraine with aura
- Other -> Please specify ____________

13. The 18-year-old male is consulted for 5 similar episodes in 5 years. During these episodes, he has blurred vision, followed by tingling of one hand and one side of the face, during 2 of such episodes his language was also impaired. All untreated symptoms last up to 1 hour. These episodes were either not followed by any headache or followed by headaches of a minor intensity (2-3/10) without photo-/phonophobia or nausea. The patient has no other known diseases. Physical examination and brain imaging is normal. Indicate the most likely diagnosis:

- Tension-type headache
- Somatoform disorder
- Recurrent transient ischemic attacks
- Migraine with aura
- Other -> please specify ____________

14. The 50-year-old female has suffered recurrent attacks of severe pulsating headaches with need for rest, nausea, and frequent vomiting since 20 years old. During the last 15 years her condition is gradually worsening and currently she has headaches almost every day. About 15 days per month the headache is very severe, pulsating and accompanied with nausea, aggravated by routine physical activity. During these attacks she takes Citramon (NSAID + caffeine). On the remaining days the headache is moderate, without any accompanying symptoms and no medication. The patient has elevated blood pressure since 40 years old, controlled with medications. She also has a known 5-year-long history of anxiety and sleep disorder, controlled with medications. Physical examination and brain imaging is normal. Indicate the most likely diagnosis:

- Tension-type headache
- Mixed headache: migraine and tension-type headache
- Chronic migraine with a medication overuse headache
- Vascular headache
- Other -> please specify ____________

15. What percentage of migraine patients do you prescribe triptans for attack treatment?

- I do not prescribe triptans for attack treatment
- 1-10%
- 11-25%
- 26-50%
- 51-75%
- 76-90%
- 91-100%

16. What percentage of migraine patients do you prescribe oral preventive medications?

- I do not prescribe oral preventive medications→ 19 question
- 1-10%
- 11-25%
- 26-50%
- 51-75%
- 76-90%
- 91-100%

17. Indicate 3 oral medications and dietary supplements which you prescribe most frequently as preventive treatment for migraine:

- Propranolol
- Metoprolol
- Nebivolol
- Candesartan
- Lisinopril
- Topiramate
- Valproic acid
- Amitriptyline
- Nortriptyline
- Venlafaxine
- Magnesium citrate

- Coenzyme Q10
- Other

17.1 Please specify your level of agreement or disagreement on a five-level Likert scale for statements about efficacy and safety of preventive treatment for migraine (5 - strongly agree, 1 - strongly disagree). (→ 21)

1. Prescribed oral medications and dietary supplements are an effective option for preventive migraine treatment (Likert scale).
    - Strongly agree (5)
    - Agree (4)
    - Neither agree nor disagree (3)
    - Disagree (2)
    - Strongly disagree (1)
2. Prescribed oral medications and dietary supplements are a safe option for preventive migraine treatment (Likert scale).
    - Strongly agree (5)
    - Agree (4)
    - Neither agree nor disagree (3)
    - Disagree (2)
    - Strongly disagree (1)

18. Indicate oral medications and dietary supplements which you have never prescribed as preventive treatment for migraine (select all that apply):

- Propranolol
- Metoprolol
- Nebivolol
- Candesartan
- Lisinopril
- Topiramate
- Valproic acid
- Amitriptyline
- Nortriptyline
- Venlafaxine
- Magnesium citrate
- Coenzyme Q10
- I have prescribed all mentioned medications and dietary supplements

19. What is your personal experience with prescribing non-pharmacological neuromodulation (e.g. external trigeminal nerve stimulation, Cefaly) for the treatment of migraine?

- I prescribe this device for the migraine treatment → 19.1, 19.2
- I do not prescribe this device for the migraine treatment → 19.3

19.1 Please indicate the number of your migraine patients who have been prescribed non-pharmacological neuromodulation during the last year ______________

19.2 Please specify your level of agreement or disagreement on a five-level Likert scale for statements about efficacy and safety of non-pharmacological neuromodulation for the treatment of migraine (5 - strongly agree, 1 - strongly disagree): (→ 20)

1. Non-pharmacological neuromodulation is an effective option for the treatment of migraine (Likert scale):
    - Strongly agree (5)
    - Agree (4)
    - Neither agree nor disagree (3)
    - Disagree (2)
    - Strongly disagree (1)
2. Non-pharmacological neuromodulation is a safe option for the treatment of migraine (Likert scale):
    - Strongly agree (5)
    - Agree (4)
    - Neither agree nor disagree (3)
    - Disagree (2)
    - Strongly disagree (1)

19.3 Please specify (select all that apply):

- I do not prescribe this device because I do not have eligible patients
- I do not prescribe this device because I do not have experience with this option of treatment
- I do not prescribe this device because I doubt the efficacy of this option of treatment
- I do not prescribe this device because I doubt the safety of this option of treatment
- I do not prescribe this device because this option of treatment has no reimbursement *(N.B. Reimbursement differs in Baltic countries)*
- Other

20. What is your personal experience with prescribing onabotulinumtoxinA for the chronic migraine treatment?

- I prescribe this option of migraine treatment → 20.1, 20.2, 20.3
- I do not prescribe this option of migraine treatment → 20.4

20.1 Please specify (select all that apply):

- I prescribe and inject myself onabotulinumtoxinA according to the PREEMPT protocol
- I prescribe and inject myself onabotulinumtoxinA not according to the PREEMPT protocol
- I prescribe onabotulinumtoxinA but I do not perform the procedure myself

20.2 Please indicate the number of your migraine patients who have been prescribed onabotulinumtoxinA during the last year _______________

20.3 Please specify your level of agreement or disagreement on a five-level Likert scale for statements about efficacy and safety of treatment with onabotulinumtoxinA (5 - strongly agree, 1 - strongly disagree). (→ 21)

3. Treatment with onabotulinumtoxinA is an effective option for chronic migraine treatment (Likert scale).
    - Strongly agree (5)

- Agree (4)
- Neither agree nor disagree (3)
- Disagree (2)
- Strongly disagree (1)

4. Treatment with onabotulinumtoxinA is a safe option for chronic migraine treatment (Likert scale).
    - Strongly agree (5)
    - Agree (4)
    - Neither agree nor disagree (3)
    - Disagree (2)
    - Strongly disagree (1)

20.4 Please specify (select all that apply):

- I do not prescribe onabotulinumtoxinA because I do not have eligible patients
- I do not prescribe onabotulinumtoxinA because I do not have experience with this option of treatment
- I do not prescribe onabotulinumtoxinA because I doubt the efficacy of this option of treatment
- I do not prescribe onabotulinumtoxinA because I doubt the safety of this option of treatment
- I do not prescribe onabotulinumtoxinA because this option of treatment has no reimbursement *(N.B. Reimbursement differs in Baltic countries)*
- Other

21. What is your personal experience with prescribing anti-CGRP monoclonal antibodies for migraine prevention?

- I prescribe these medicines for migraine prevention → 21.1, 21.2, 21.3
- I do not prescribe these medicines for migraine prevention → 21.4

21.1 Please indicate all medications which you have ever prescribed (select all that apply):

- Erenumab
- Fremanezumab
- Galcanezumab
- Eptinezumab

21.2 Please indicate the number of your migraine patients who have been prescribed anti-CGRP monoclonal antibodies for migraine prevention during the last year _______________

21.3 Please specify your level of agreement or disagreement on a five-level Likert scale for statements about efficacy and safety of anti-CGRP monoclonal antibodies (5 - strongly agree, 1 - strongly disagree): (→ 22)

1. Anti-CGRP monoclonal antibodies are effective medications for the treatment of migraine (Likert scale):
    - Strongly agree (5)
    - Agree (4)
    - Neither agree nor disagree (3)
    - Disagree (2)
    - Strongly disagree (1)

3. Anti-CGRP monoclonal antibodies are safe medications for the treatment of migraine (Likert scale):
    - Strongly agree (5)
    - Agree (4)
    - Neither agree nor disagree (3)
    - Disagree (2)
    - Strongly disagree (1)

21.4 Please specify (select all that apply):
- I do not prescribe these medicines because I do not have eligible patients
- I do not prescribe these medicines because I do not have experience with this type of medication
- I do not prescribe these medicines because I doubt the efficacy of this type of medication
- I do not prescribe these medicines because I doubt the safety of this type of medication
- I do not prescribe these medicines because this type of medication has no reimbursement *(N.B. Reimbursement differs in Baltic countries)*
- Other

22. Which tools do you use to assess the efficacy of migraine prevention treatment? Select all that apply.
- Patient's impression and satisfaction
- Headache diary/calendar
- Disability and/or other headache specific scales (MIDAS, HIT-6, HALT, HURT questionnaires)
- Other

23.
- I confirm that the answers to the survey were given on a voluntary basis and I agree with the use of my anonymous answers for the research.

____
